# Bioinformatics identification of mitochondria and macrophage polarization-related genes in COPD and their potential mechanisms

**DOI:** 10.3389/fimmu.2025.1675292

**Published:** 2025-10-08

**Authors:** Chenchen Zhang, Peishu Fu, Juanchun Yu, Lingling Liu, Xiong Wei, Xiao Li

**Affiliations:** ^1^ Department of Geriatrics, Chongqing Key Laboratory of Aging and Regeneration Medicine, The First Affiliated Hospital (Southwest Hospital) of Army Medical University, Chongqing, China; ^2^ Department of Pharmacy, the First Affiliated Hospital of Army Medical University, Chongqing, China; ^3^ Department of Clinical Laboratory, the Second Affiliated Hospital of Army Medical University, Chongqing, China

**Keywords:** bioinformatics, COPD, macrophage polarization, mitochondria, P2RY1, UBASH3B, HMCN1

## Abstract

**Introduction:**

This study investigated key genes associated with both chronic obstructive pulmonary disease (COPD) and macrophage polarization or mitochondrial dysfunction, and explored their underlying mechanisms through bioinformatics analysis.

**Methods:**

Data from GSE151052, GSE106986, and GSE171541 were utilized. Critical module genes linked to mitochondria-related genes (MRGs) and macrophage polarization-related genes (MPRGs) were identified *via* co-expression networks. Biomarkers for COPD were then screened using differential expression analysis, machine learning, and receiver operating characteristic (ROC) curves. A nomogram was developed to assess COPD risk. Additionally, immune infiltration, molecular regulation, and drug prediction analyses were conducted. Single-cell analysis in GSE171541 identified key cell types involved in COPD.

**Results:**

A series of analyses identified three COPD biomarkers—P2RY1, UBASH3B, and HMCN1—which exhibited strong discriminatory power between COPD and control samples. The nomogram effectively predicted COPD risk. Immune infiltration analysis revealed a strong positive correlation between UBASH3B and immature dendritic cells, while P2RY1 showed a strong negative correlation with eosinophils. Molecular regulation indicated that all three biomarkers were modulated by specific miRNAs and transcription factors. Nickel was identified as a potential drug co-predicted for the biomarkers. Single-cell analysis identified seven key cell types: macrophages, monocytes, T cells, AT2 cells, proliferating cells, endothelial cells, and stromal cells.

**Conclusion:**

Three biomarkers associated with mitochondrial function and macrophage polarization were identified in COPD through bioinformatics analysis. These biomarkers offer potential for enhancing COPD diagnosis and treatment, and provide insights into the molecular mechanisms underlying the disease.

## 1 Introduction

Chronic obstructive pulmonary disease (COPD) is a chronic respiratory condition marked by persistent airway inflammation due to exposure to toxic particles or gases (1). According to the latest data from the Global Burden of Disease (GBD) study, COPD has become the third leading cause of death worldwide and ranked sixth globally in terms of disability-adjusted life years (DALYs) in 2022 (2). Particularly concerning is the projection that by 2050, COPD will rise to the fourth position in global DALYs, second only to ischemic heart disease, stroke, and diabetes. This disease burden is particularly prominent in low- and middle-income countries (LMICs), especially in sub-Saharan Africa and South Asia. Due to exposure to biomass fuels, widespread tobacco use, and inadequate healthcare resources, the incidence and mortality rates of COPD in these regions are significantly higher than those in high-income countries (3). Common symptoms include wheezing, shortness of breath, chest tightness, and difficulty breathing (4, 5). The pathogenesis of COPD involves inflammation, oxidative stress, antiprotease imbalance, muscle dysfunction, and alterations in lung microbiota (5–7). Emphysema, a hallmark of COPD, is characterized by reduced elasticity in the distal airways, airway overexpansion, increased lung capacity, and airway damage (8). Proteases contribute to lung tissue destruction, impairing tissue remodeling and repair, while antiproteases protect lung tissue by inhibiting protease activity. The development of emphysema is believed to stem from an imbalance between proteases and antiproteases, making it critical to explore the relationship between COPD and these proteins (9).

Macrophages are key immune cells involved in antigen presentation, phagocytosis, and the release of cytokines and proteases, playing a central role in immune regulation (10). They contribute to lung inflammation and tissue remodeling by releasing various proteases (e.g., MMP-12) and pro-inflammatory factors (e.g., IL-6, TNF-α) (11, 12). Smoking-induced inflammation activates macrophages, prompting the release of excessive proteases that further damage lung structure (13). In addition to their immune functions, macrophages clear pathogens and cellular debris through phagocytosis. However, macrophage dysfunction can exacerbate inflammation and worsen COPD (11, 12, 14). Macrophage polarization refers to the changes in morphology and function that occur in response to environmental stimuli. The immunophenotype and function of alveolar macrophages are largely influenced by the local microenvironment of the alveolar space (15, 16). To maintain homeostasis, the immune response undergoes dynamic changes during disease progression, with alveolar macrophages, like other macrophages, exhibiting polarization (15). Despite these observations, the relationship between macrophage polarization and COPD emphysema remains unclear and warrants further investigation.

Mitochondria are organelles responsible for regulating energy and oxidative metabolism, cellular respiration, inflammation, and cell death (17). Mitochondrial dysfunction and cellular aging are key features in the pathogenesis of COPD (17). Previous studies have demonstrated that mitochondrial peptides are altered in stable COPD and are associated with various disease characteristics. Mitochondria play a significant role in oxidative stress responses and apoptotic processes (18, 19). Harmful factors such as smoking lead to mitochondrial dysfunction and the production of reactive oxygen species (ROS), triggering oxidative stress and cellular damage. Oxidative stress not only directly damages lung tissue but also amplifies inflammatory responses and protease release through the activation of inflammatory signaling pathways (e.g., NF-κB) (18, 19). In COPD pathology, complex interactions between proteases, macrophages, and mitochondria are evident. External factors like smoking activate macrophages, prompting them to release proteases and initiate an inflammatory response. Simultaneously, oxidative stress from mitochondrial dysfunction exacerbates inflammation, further promoting protease release. Additionally, protease activity degrades the extracellular matrix (ECM) in the lungs, contributing to alveolar wall damage and emphysema formation (20–22). However, research on the mechanisms involving these proteins in COPD remains limited (23). The genes linking macrophage polarization and mitochondrial function in COPD are not well understood, highlighting the need for further investigation of these gene sets in COPD studies.

Thus, this study aims to utilize bioinformatics methods to explore key genes associated with COPD, macrophage polarization, and mitochondrial dysfunction, as well as their potential mechanisms. By predicting drugs, identifying candidate treatments, and using single-cell sequencing to assess gene expression at the cellular level, this study offers a new perspective for understanding the impact of macrophage polarization and mitochondrial-related genes in COPD.

## 2 Materials and methods

### 2.1 Data collection

The COPD-related datasets were retrieved from the Gene expression omnibus (GEO) database (https://www.ncbi.nlm.nih.gov/geo/). GSE151052 (platform: GPL17556) contained lung tissue samples from 77 COPD and 40 control subjects, while GSE106986 (platform: GPL13497) included samples from 14 COPD and 5 control subjects (**Supplementary Table 1**). The single-cell dataset, GSE171541 (platform: GPL24676), comprised lung tissue samples from 3 COPD and 6 control subjects. Additionally, 1,136 mitochondrion-related genes (MRGs) (24) and 35 macrophage polarization-related genes (MPRGs) (25) were sourced from previous studies.

### 2.2 Weighted gene co-expression network analysis

To identify genes associated with MRGs and MPRGs in GSE151052, WGCNA (26) (v1.72-1) was performed to construct a co-expression network and screen critical module genes. First, the single-sample gene set enrichment analysis (ssGSEA) algorithm in GSVA (27) (v1.48.3) was applied to calculate the MRG and MPRG scores for all samples in GSE151052, followed by the use of the Wilcoxon test to compare these scores between COPD and control samples. Next, the WGCNA (26) (v1.72-1) package was used to perform hierarchical clustering on the GSE151052 samples, and outliers were removed. An appropriate soft threshold was selected to construct the scale-free network, ensuring the R2 was close to 0.8 and the mean connectivity approached zero. A systematic clustering tree was then used to classify genes into different modules, applying dynamic tree cutting and setting the minimum number of genes per module to 300. MRG and MPRG scores were considered as traits, and Pearson correlation analysis was performed to explore the relationship between traits and modules. The module with the highest correlation (|r| > 0.3, p < 0.05) was selected as the key module, and the genes within it were defined as critical module genes.

### 2.3 Differential expression and enrichment analysis

To screen for differentially expressed genes (DEGs) between COPD and control groups and explore their functional characteristics, the limma (28) (v3.56.2)was used to perform differential expression analyses on the GSE151052 and GSE106986 datasets, respectively. The screening criteria were set as |log2FC| > 0.5 and P.adjust < 0.05, and corresponding DEGs for the two datasets (designated as DEGs1 and DEGs2) were obtained respectively. Subsequently, the ggplot2 (29) (v3.4.2) was employed to generate volcano plots, which visually displayed the degree of differential gene expression. Meanwhile, the circlize (30) (v0.4.15) was used to plot expression heatmaps of the top 10 genes with the largest fold changes among upregulated and downregulated genes in each dataset, so as to present the expression patterns of core DEGs in samples. Common upregulated and downregulated genes were identified by overlapping the up- and down-regulated DEGs, respectively, resulting in common DEGs. Further, the eulerr (31) package (v7.0.0) was utilized to construct Venn diagrams, and intersection analysis was performed between the aforementioned shared DEGs and key module genes, thereby screening out candidate genes. Finally, to explore the common functions and related signaling pathways among the candidate genes, the clusterProfiler (32) (v4.8.2) was adopted to conduct enrichment analyses, including Gene ontology (GO) and Kyoto encyclopedia of genes and genomes (KEGG). With a screening threshold of p < 0.05, the functional terms and pathways significantly enriched in the candidate genes were identified.

### 2.4 Machine learning

To further identify biomarkers for COPD, machine learning techniques were applied. In GSE151052, candidate genes were subjected to least absolute shrinkage and selection operator (LASSO) analysis using glmnet (33) (v4.1-6) to identify signature genes through 10-fold cross-validation. Additionally, Support vector machine recursive feature elimination (SVM-RFE) was performed using e1071 (v1.7-13 https://CRAN.R-project.org/package=e1071) to select the gene combination with the lowest error rate as the optimal set of signature genes. The signature genes were then intersected to identify candidate biomarkers. Finally, Support vector machine recursive feature elimination (ROC) curves were generated using pROC (34) (1.18.4) in GSE151052 and GSE106986 to assess the discriminatory ability of the candidate biomarkers between COPD and control samples, and their generalizability. An area under the curve (AUC) greater than 0.7 was considered indicative of a substantial discriminatory ability, and genes meeting this threshold were designated as biomarkers in this study.

### 2.5 Construction and evaluation of the nomogram

To evaluate the predictive value of biomarkers for the risk of COPD, in the GSE151052 dataset, based on the expression levels of biomarker, the rms (35) (v6.7-0) was used to perform parameter estimation via maximum likelihood estimation, and a nomogram risk prediction model was constructed. Based on this model, each biomarker was independently evaluated, and each biomarker was assigned a specific numerical point value. The total score was calculated by summing the scores of all biomarkers, with a higher score indicating a higher risk of developing COPD. Subsequently, the pROC (36) package (v1.18.4) was used to plot ROC curves in the GSE151052 and GSE106986 datasets respectively, so as to evaluate the predictive performance of the nomogram model. Among them, the AUC was used as the main evaluation index; a higher AUC value indicated a better predictive effect of the model, with an AUC > 0.7 considered as the criterion for favorable predictive performance.

### 2.6 Immune infiltration analysis

To investigate the role of biomarkers in the immune microenvironment of COPD, infiltration analyses of 28 immune cell types were performed. Enrichment scores for these immune cells in COPD and control samples were calculated using the ssGSEA algorithm. Differences in infiltration levels between the groups were assessed using the Wilcoxon test. Spearman correlation analysis was then used to examine relationships between differential immune cells, as well as between biomarkers and differential immune cells, using the psych package (37) (v2.3.6).

### 2.7 Construction of biomarlers related networks

To investigate the mechanism of action and potential regulatory relationships of biomarkers, genes associated with biomarker function were predicted using GeneMANIA database (https://genemania.org/), and gene-gene interaction (GGI) networks were constructed. Then, in order to explore the upstream regulatory mechanisms of biomarkers in COPD, relevant transcription factors (TF) that could regulate the biomarkers were predicted using the NetworkAnalyst platform (https://www.networkanalyst.ca/) based on the JASPAR database. Subsequently, microRNAs (miRNAs) targeting the biomarkers were predicted by the miRDB database (http://www.mirdb.org/). Following these predictions, both the TF-mRNA and miRNA-mRNA networks were constructed using Cytoscape (38) (v3.9.1). respectively.

### 2.8 Analysis of the interaction between biomarkers and COPD treatment drugs

To investigate the interaction between biomarkers and COPD therapeutic drugs, Albuterol—a bronchodilator commonly used in clinical practice—was chosen as the research subject. Its pharmacological effects had been widely confirmed in the management of acute COPD symptoms (39). The 3D structures of the biomarker proteins were downloaded from the Protein Data Bank (PDB, https://www.rcsb.org/), and the 3D structure of the Albuterol molecular ligand (the key active component) was retrieved from the PubChem database (https://pubchem.ncbi.nlm.nih.gov/). The obtained proteins and ligands were uploaded to the CB-Dock2 online platform (https://cadd.labshare.cn/cb-dock2/php/index.php) for molecular docking, and the binding free energy was calculated. Generally, a smaller binding free energy indicated a stronger binding ability; specifically, a binding free energy of ≤-5.0 kcal/mol was regarded as representing a tight binding relationship. To further verify the reliability of the interaction, Albuterol and known COPD therapeutic targets (e.g., IL17, MMP9) were subjected to comparative molecular docking analysis. This analysis was conducted to evaluate the potential clinical significance of the binding between the biomarker and the drug.

### 2.9 Single-cell RNA sequencing analysis

Single-cell samples were filtered using Seurat (40) (v4.3.0) with the following criteria: genes detected in fewer than 200 cells were excluded, along with cells where nFeature_RNA (number of genes per cell) was < 7000, nCount_RNA (number of counts per cell) was < 50,000, or percent.mt (mitochondrial proportion of expressed genes) was ≥ 20%. The remaining cells and genes were selected for further analysis. The top 2,000 highly variable genes were identified using vst, followed by PCA to select appropriate principal components (PCs) for subsequent analyses. Unsupervised clustering was then performed using FindNeighbors and FindClusters functions (resolution = 0.1), and cells were grouped using UMAP. Cell types were annotated based on marker genes. CellChat (41) (v1.6.1) was employed to explore intercellular communication and interactions. To examine the distribution of biomarkers across cellular taxa in COPD and control samples, Wilcoxon testing was used to assess differences in biomarker expression between cell groups, with significant differences identifying key cells. Additionally, Monocle (42) (v2.26.0) was applied to explore the developmental and differentiation trajectories of the key cells. The input data were the raw UMI count matrices and metadata of each cell subset that had been preprocessed by Seurat (40) (v4.3.0). During the analysis, expressionFamily = negbinomial.size() was specified to model the distribution of UMI data, with lowerDetectionLimit set to 0.5. After data normalization was conducted using estimateSizeFactors and gene dispersion was estimated via estimateDispersions, the detectGenes() function was applied to retain genes with an expression level > 0.1 in ≥ 10% of cells (with min_expr = 0.1). The 2000 highly variable genes screened by Seurat were used as the ordering gene set. Subsequently, the DDRTree algorithm was employed to reduce the data dimension to 2D, and orderCells was used to construct a minimum spanning tree and calculate pseudotime. Finally, plot_cell_trajectory (colored by pseudotime/cell state) and plot_genes_in_pseudotime (to display the expression dynamics of biomarkers) were respectively used to visualize the results.

To further investigate the macrophage polarization status and the association between gene expression and polarization phenotypes, studies were conducted on macrophages. The R package Seurat (40) (v4.3.0) was employed: first, the RunPCA function was used for principal component dimensionality reduction; subsequently, the FindNeighbors and FindClusters functions (with the resolution parameter set to 0.1) were applied for cell clustering. The obtained cell clusters were then annotated using marker genes reported in the literature (43–45) to identify different cell subpopulations. To clarify the expression characteristics of biomarkers in the single-cell dataset, bubble plots were used to visually display the expression distribution of these genes across different cell types. In addition, to reveal the biological functions and pathways involved in each cell type, the ReactomeGSA (46) (v1.21.1) package was utilized to perform functional enrichment analysis for each annotated cell type. The top 10 differentially ranked functional items were screened out to reveal differences in biological functions among different cell types.

### 2.10 Reverse transcription quantitative real-time PCR

In the study, whole blood samples from patients with COPD (n = 5) and healthy controls (n = 5) were collected from the First Affiliated Hospital of Army Medical University of the Chinese People’s Liberation Army. Total RNA from synovial tissue was extracted using Trizol (Ambion), followed by reverse transcription into complementary DNA (cDNA) using the SweScript First Strand cDNA Synthesis Kit. RT-qPCR was performed using the 2xUniversal Blue SYBR Green qPCR Master Mix. The primers for biomarkers were synthesized by Beijing Tsingke Biotech Co., Ltd. (Beijing, China), and the sequences of primers used in RT-qPCR are listed in **Supplementary Table 2**. GAPDH served as the internal reference gene. Each biological sample was tested in triplicate. The study was approved by the Ethics Committee of the First Affiliated Hospital of Army Medical University of the Chinese People’s Liberation Army (No.: KY2024142), and the patient/subject has signed a written informed consent to participate in this research.

### 2.11 Statistical analysis

All analyses were conducted using R. Statistical significance was defined as P < 0.05.

## 3 Result

### 3.1 Identification of 2,837 critical module genes related to MRGs and MPRGs scores

**Figure 1:**
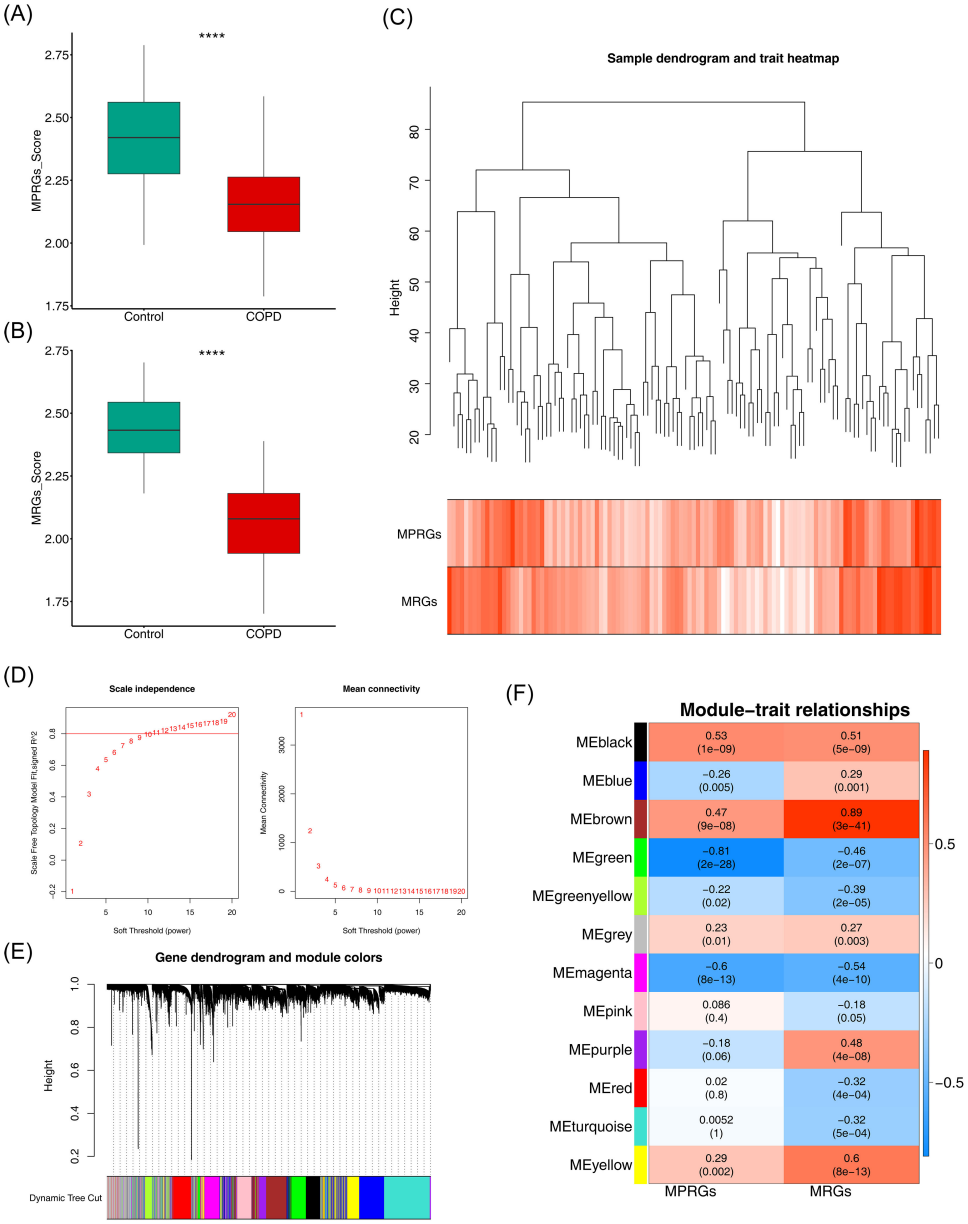
Identification of mitochondria and macrophage polarization-related module genes. **(A, B)** Box plots of intergroup differences in MRGs score and MPRGs score. ****, p<0.0001. **(C)** Hierarchical clustering plot of samples. Branches represent samples, and the ordinate represents the height of hierarchical clustering. **(D)** Soft thresholding filtering results. **(E)** Module dynamic cutting Tree. **(F)** Heatmap of correlation between modules and core traits.

The MRGs and MPRGs scores were significantly lower in the COPD group (p < 0.05), indicating a potential association with COPD (**Figures 1A, B**). All samples in GSE151052 were clustered, with no outlier samples identified (**Figure 1C**). A soft threshold of 11 was selected to construct the scale-free network (**Figure 1D**). The systematic phylogenetic tree was categorized into 12 modules using dynamic tree cutting (**Figure 1E**). Among these, the brown (r = 0.89, p = 3 × 10^–41^) and green (r = -0.81, p = 2 × 10^–28^) modules exhibited the strongest correlations with MRGs and MPRGs scores, respectively. The 2,837 genes within these modules were defined as critical module genes (**Figure 1F**).

### 3.2 The 17 candidate genes involved in complex pathways and functions

**Figure 2:**
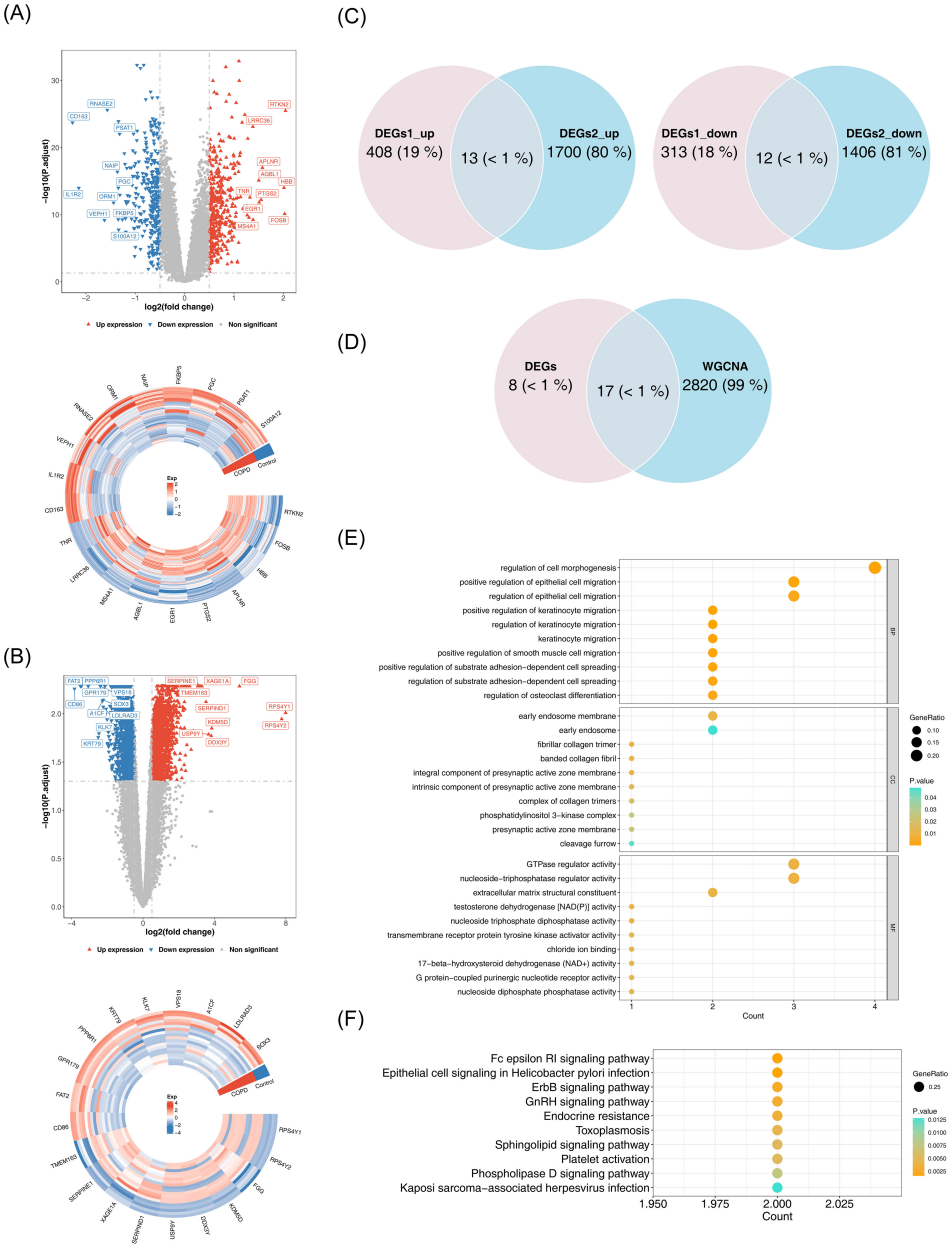
Identification and functional analysis of candidate genes. **(A)** Volcano plot and heatmap of differential analysis. Left panel: Red upward triangles represent upregulated differentially expressed genes; blue downward triangles represent downregulated genes; gray points indicate non-significant genes. Right panel: Gene names are labeled outside the circle. Colors indicate normalized gene expression levels: red indicates high expression, blue indicates low expression. **(B)** Volcano plot and heatmap of differential analysis. Left panel: Red upward triangles represent upregulated differentially expressed genes; blue downward triangles represent downregulated genes; gray points indicate non-significant genes. Right panel: Gene names are labeled outside the circle. Colors indicate normalized gene expression levels: red indicates high expression, blue indicates low expression. **(C)** Venn diagram illustrating the intersection of DEGs1 and DEGs2. **(D)** Venn diagram for candidate gene identification. **(E)** GO enrichment analysis. The horizontal axis represents the number of enriched genes, the vertical axis shows the term names, and the color of the dots indicates the significance level. **(F)** KEGG enrichment analysis.

A total of 746 DEGs1 (421 upregulated, 325 downregulated) and 3,131 DEGs2 (1,713 upregulated, 1,418 downregulated) were identified between COPD and control samples (**Figures 2A, B**). Among them, 25 common DEGs were identified, including 13 upregulated and 12 downregulated genes (**Figure 2C**). The overlap between common DEGs and critical module genes yielded 17 candidate genes (**Figure 2D**). GO analysis revealed that these genes were primarily involved in GTPase regulator activity, nucleoside-triphosphatase regulator activity, and positive regulation of smooth muscle cell migration (**Figure 2E**). KEGG pathway analysis indicated that candidate genes were associated with Fc epsilon RI signaling, ErbB signaling, GnRH signaling, and phospholipase D signaling pathways (**Figure 2F**).

### 3.3 Identification of the biomarkers

**Figure 3:**
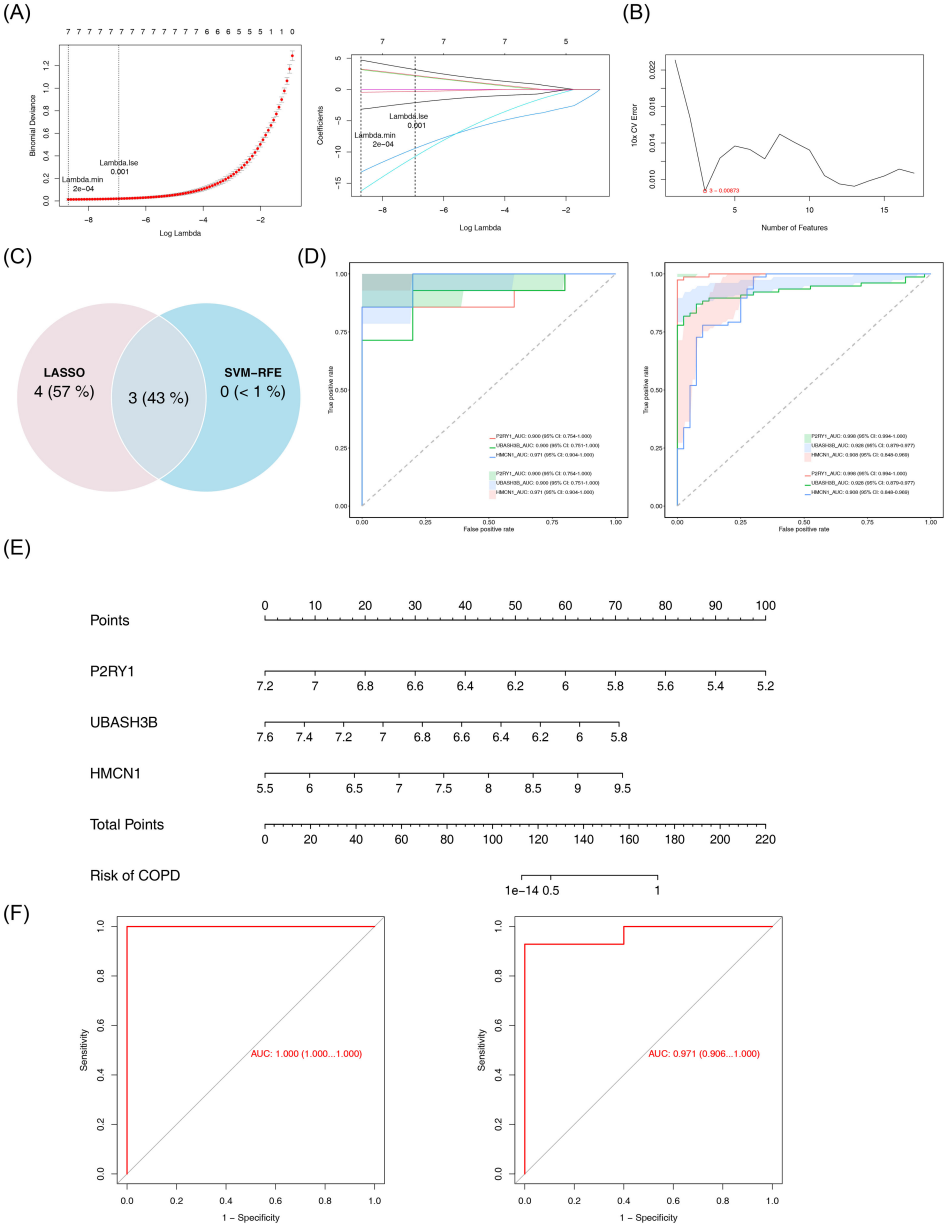
Identification and validation of biomarkers. **(A)** LASSO regression analysis for feature selection. Left panel: Binomial deviance plot as a function of log-transformed lambda, with vertical dashed lines indicating lambda.min (2×10^-4^) and lambda.1se (0.001). Right panel: Coefficient path plot of LASSO logistic regression. The abscissa is log(lambda), and the ordinate represents gene coefficients. **(B)** SVM-RFE analysis. The abscissa represents the number of genes, and the ordinate represents the error rate. **(C)** Venn diagram of results from the two machine learning algorithms. **(D)** ROC curves for individual genes. Left panel: GSE151052 dataset; Right panel: GSE106986 dataset. **(E)** Nomogram for key genes. **(F)** Model validation ROC curve. The horizontal axis represents the false positive rate, and the vertical axis represents the true positive rate. The closer the area under the curve (AUC) is to 1, the better the model performance. Left panel: GSE151052; right panel: GSE106986.

Machine learning was used to identify candidate biomarkers. In LASSO analysis, with a Lambda.min of 2e-04, seven signature genes were selected: HMCN1, KLF10, CHN1, P2RY1, UBASH3B, DOCK5, and SIGLEC16 (**Figure 3A**). SVM-RFE analysis, with an error rate of 0.00873, selected three signature genes: P2RY1, UBASH3B, and HMCN1 (**Figure 3B**). Overlapping these results led to the identification of three candidate biomarkers (**Figure 3C**). ROC analysis showed AUC values greater than 0.9 for the three biomarkers (**Figure 3D**), indicating their strong ability to distinguish between COPD and control samples. These biomarkers were thus considered reliable for further analysis. A nomogram for the biomarkers was constructed (**Figure 3E**). Furthermore, the ROC curve of the model revealed AUC values exceeding 0.9 (**Figure 3F**), demonstrating excellent predictive performance and generalizability.

### 3.4 Immune infiltration analysis of biomarkers

**Figure 4:**
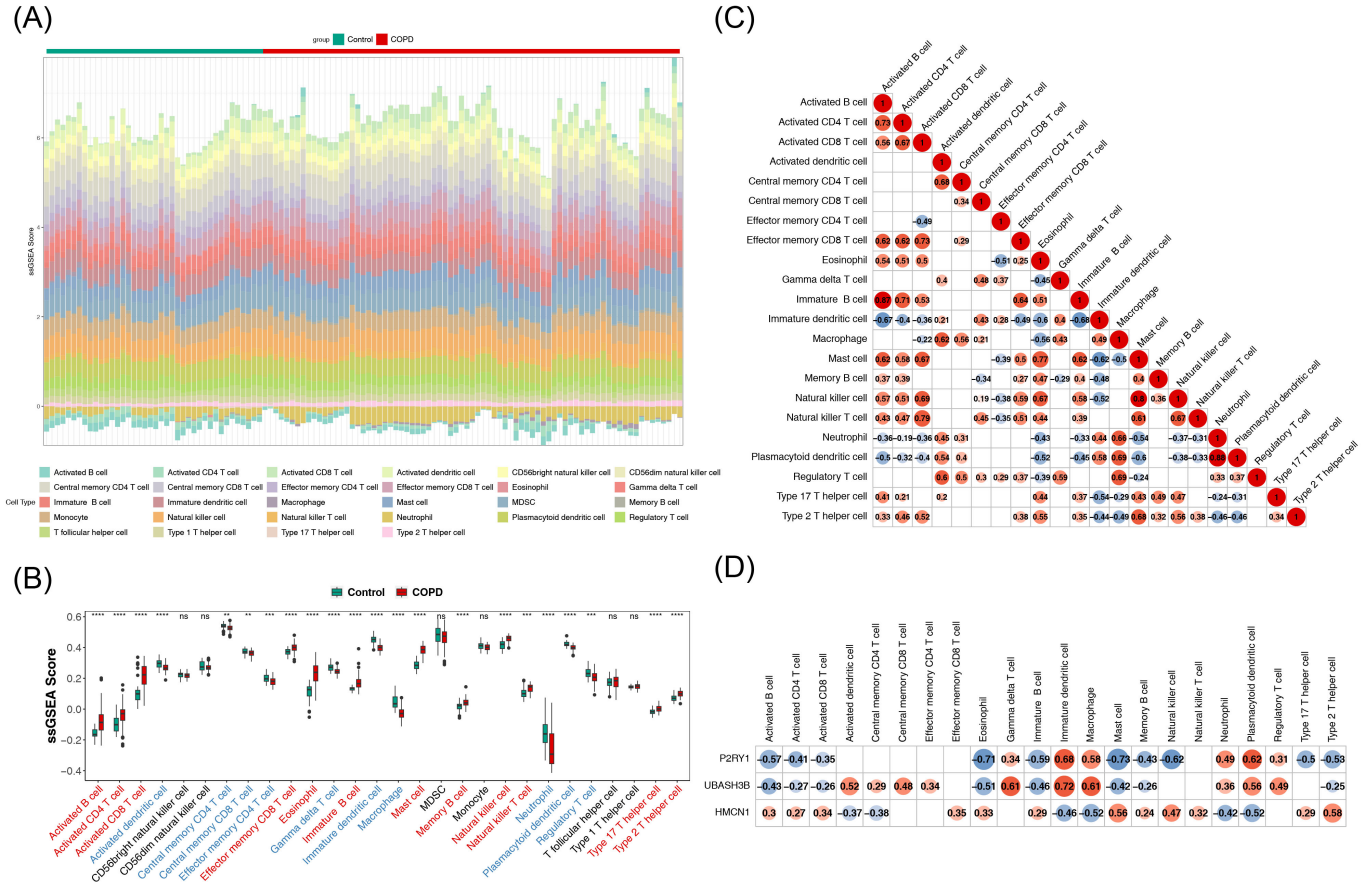
Analysis of immune infiltration of key genes. **(A)** Stacked plot showing immune cell infiltration. **(B)** Box plots depicting immune cell infiltration. The horizontal axis represents 28 immune cell types (red indicates significantly upregulated immune cells, blue indicates significantly downregulated immune cells). The vertical axis represents the score of immune cells in samples. Ns denotes non-significant; **, p < 0.01; ***, p < 0.001; ****, p < 0.0001. **(C)** Heatmap of correlations between differential immune cells. Both horizontal and vertical axes represent differential immune cells (labeled with their names). Red circles indicate positive correlations, with the intensity of color reflecting the strength of the correlation. Blue circles represent negative correlations, with deeper colors indicating stronger negative correlations. Numbers inside circles represent correlation coefficients. Blank areas indicate non-significant correlations. **(D)** Heatmap of correlations between key genes and differential immune cells. The horizontal axis represents differential immune cells (labeled with their names), and the vertical axis represents key genes (labeled with their names). Blue circles indicate negative correlations, with deeper blue indicating stronger negative correlations. Red circles indicate positive correlations, with deeper red reflecting stronger positive correlations. Numbers inside circles represent correlation coefficients. Blank areas denote non-significant correlations.

The impact of biomarkers on the immune microenvironment was further explored. **Figure 4A** illustrates the proportion of 28 immune cell types infiltrated in COPD and control samples. Among these, 22 immune cell types showed significant differences between COPD and control samples. Specifically, activated B cells, activated CD4 T cells, and activated CD8 T cells exhibited significantly higher infiltration levels in the COPD group, whereas central memory CD4 T cells, central memory CD8 T cells, and effector memory CD4 T cells showed notably lower infiltration levels (**Figure 4B**). Correlation analysis revealed the highest positive correlation between plasmacytoid dendritic cells and neutrophils (r = 0.88, p < 0.05), while the highest negative correlation was observed between immature dendritic cells and immature B cells (r = -0.68, p < 0.05) (**Figure 4C**). Furthermore, UBASH3B demonstrated the strongest positive correlation with immature dendritic cells (r = 0.72, p < 0.05), and P2RY1 exhibited the strongest negative correlation with eosinophils (r = -0.71, p < 0.05) (**Figure 4D**). These results suggest that the biomarkers may influence COPD through immune cell interactions.

### 3.5 Construction of related networks and drug prediction

**Figure 5:**
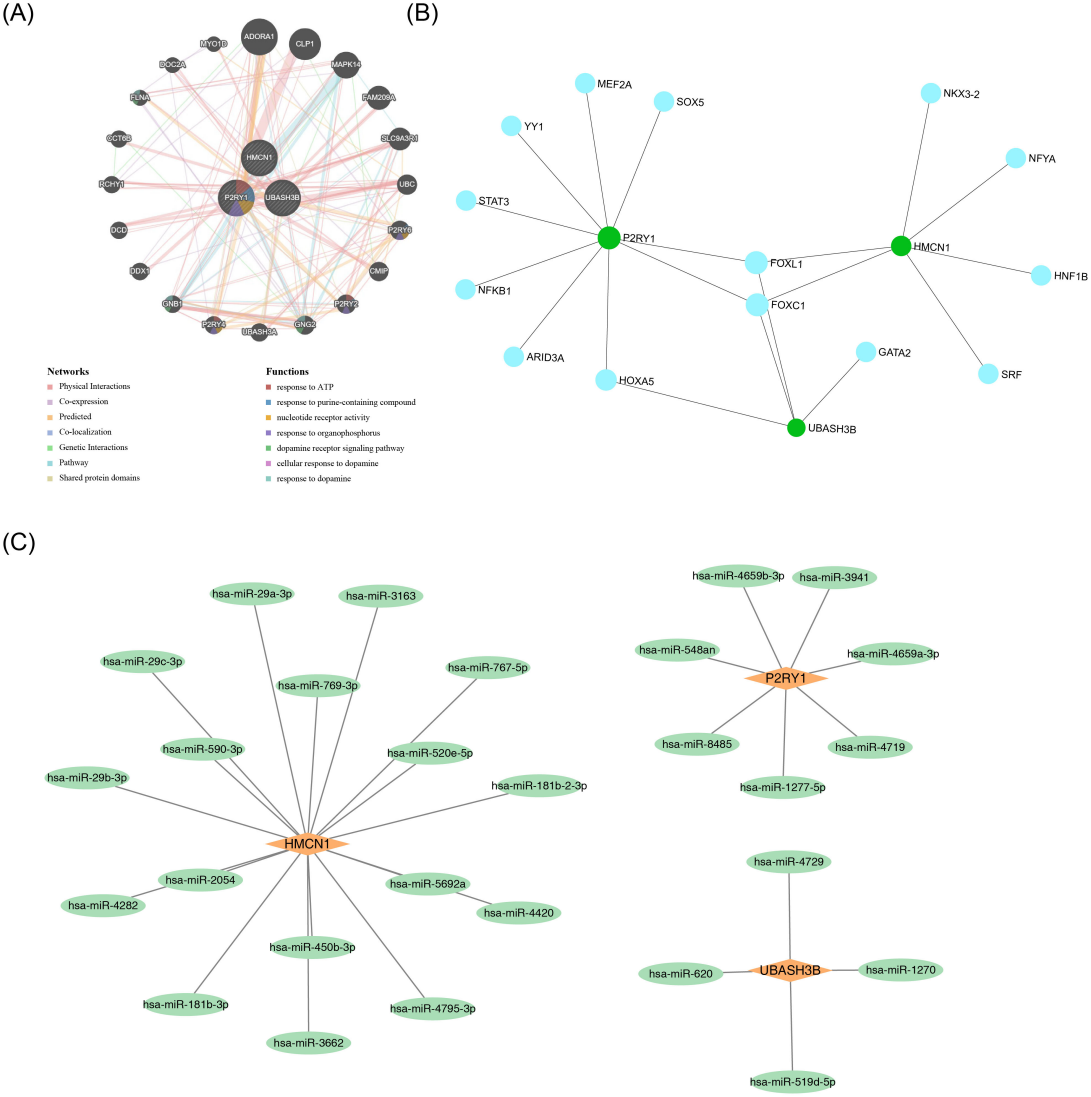
Regulatory and interaction networks of key genes. **(A)** GGI network of key genes. **(B)** TF-mRNA network. Green circles represent mRNAs, and blue circles represent predicted TFs. **(C)** miRNA-mRNA network. Orange quadrilaterals represent mRNAs, and green circles represent predicted miRNAs.

GeneMANIA analysis identified the top 20 predicted genes and the top 7 significant pathways associated with the biomarkers, revealing that they were primarily involved in responses to purine-containing compounds, nucleotide receptor activity, and related processes (**Figure 5A**). Additionally, 14 transcription factors (TFs) and 28 miRNAs were predicted through the JASPAR and miRDB databases, respectively, and TF-mRNA and miRNA-mRNA networks were constructed. All three biomarkers were found to be regulated by specific TFs and miRNAs (**Figures 5B, C**).

### 3.6 P2RY1 and UBASH3B exhibit stable binding to Albuterol

**Figure 6:**
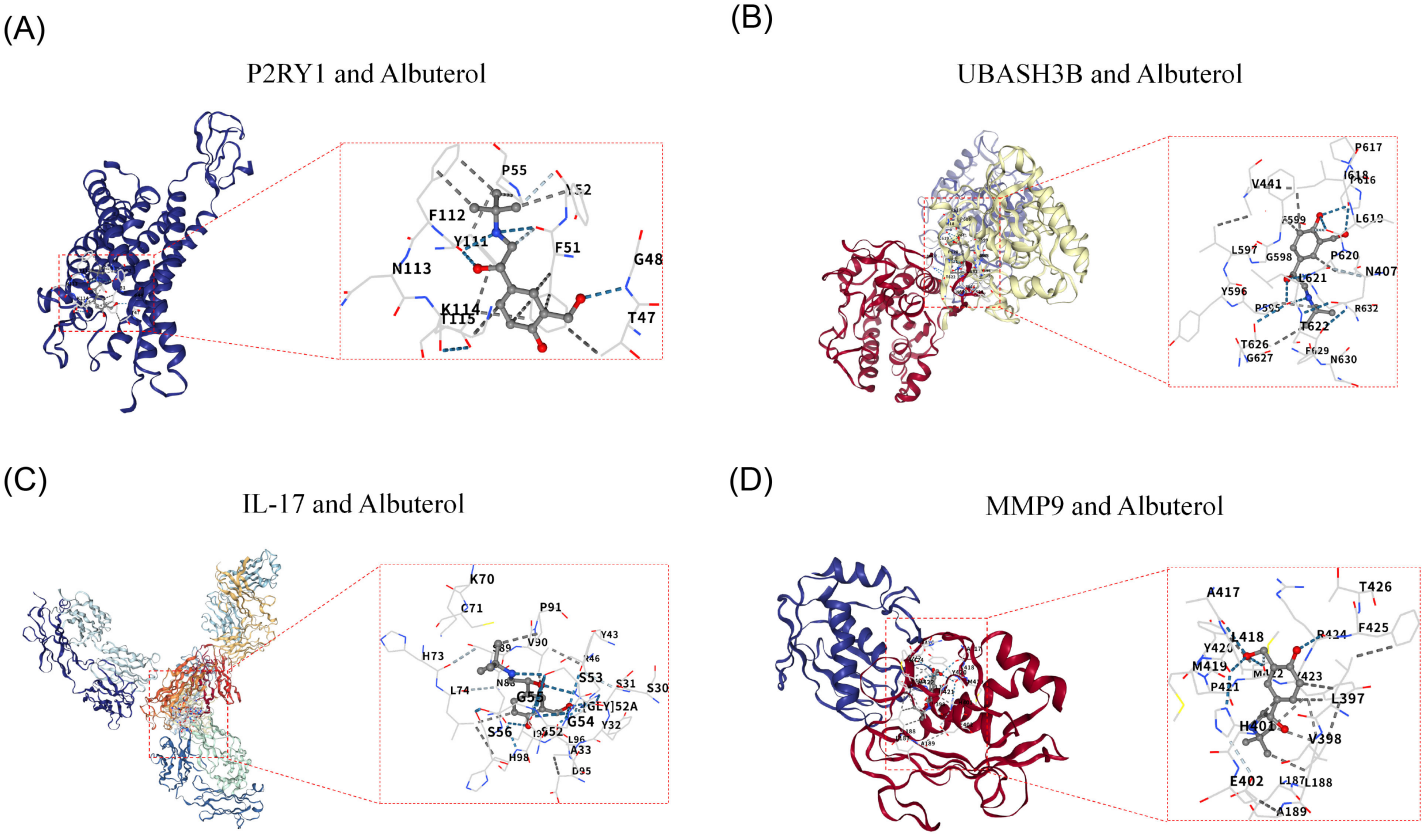
Molecular Docking and Validation of Biomarkers. **(A)** Docking Diagram of P2RY1 and Albuterol. **(B)** Docking Diagram of P2RY1 and Albuterol. **(C)** Molecular Docking Diagram of IL-17 and Drug Albuterol. **(D)** Molecular Docking Diagram of MMP9 and Drug Albuterol.

Due to the lack of a known 3D structure for HMCN1, it was not subjected to docking analysis. Further molecular docking analysis revealed that the genes P2RY1 (PDB ID: 4XNV) and UBASH3B (PDB ID: 5W5G) both exhibited strong binding ability to the drug Albuterol, with their binding free energies being -6.0 kcal/mol and -6.1 kcal/mol, respectively (**Figures 6A, B**). Both values were lower than the threshold of -5.0 kcal/mol, indicating a stable binding state. Additionally, as known therapeutic targets associated with COPD, IL-17 (PDB ID: 2VXS) and MMP9 (PDB ID: 1GKC) also had binding energies to Albuterol that were less than -5.0 kcal/mol (**Figures 6C, D**). This result was consistent with previous knowledge and validated the reliability of the molecular docking method employed in this study.

### 3.7 Annotation with 12 cell types through scRNA-seq analysis

**Figure 7:**
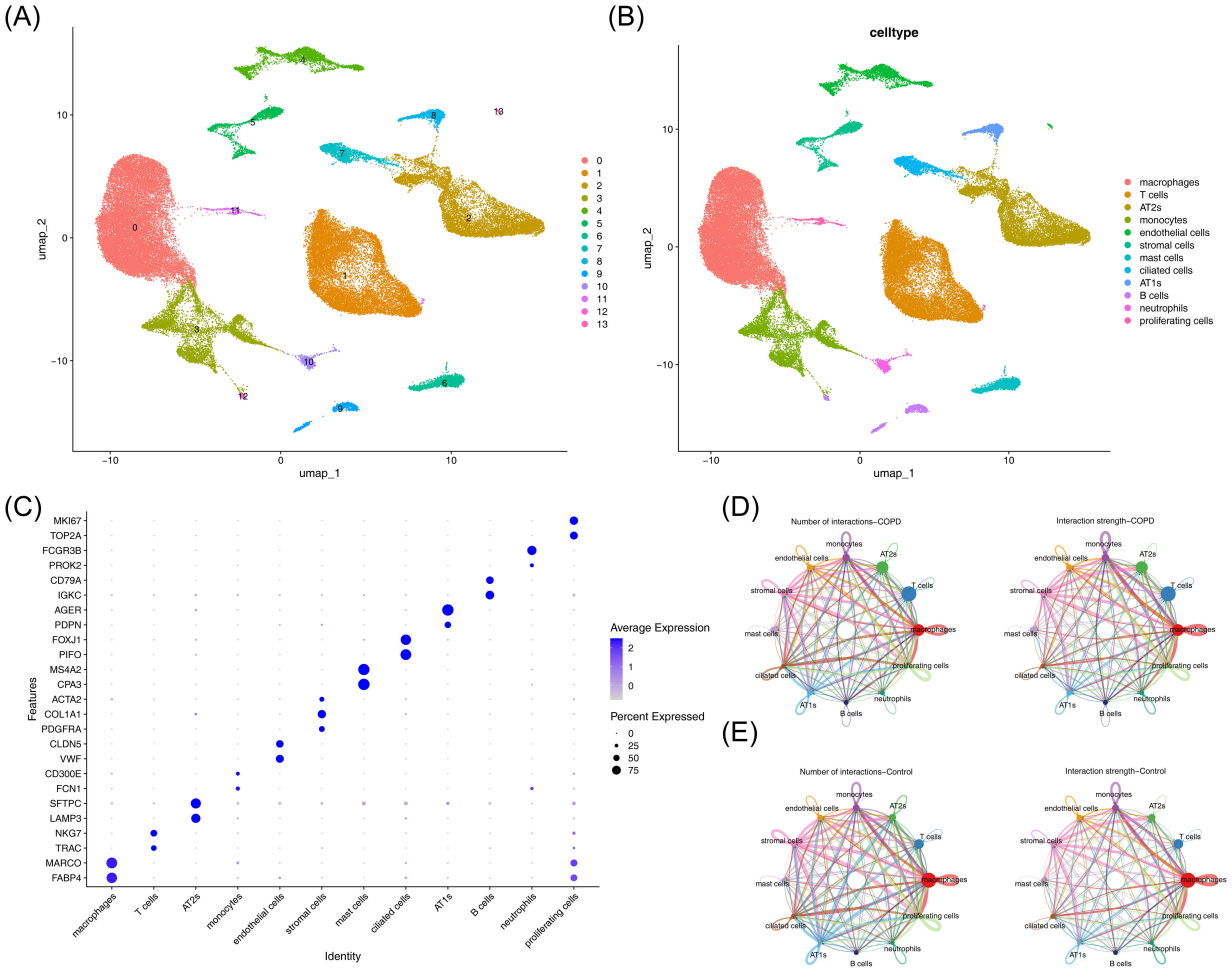
Single-cell analysis of cellular composition and intercellular communication. **(A)** UMAP clustering plot of cells. **(B)** Annotation of cell clustering subpopulations. **(C)** Additional annotation results of cell clustering subpopulations. **(D)** Cell communication network in the control group. Left panel: Interaction frequency between cells; right panel: Interaction intensity between cells. **(E)** Cell communication network in the COPD group. Left panel: Interaction frequency between cells; right panel: Interaction intensity between cells.

After filtering the GSE171541 dataset, 59,322 cells and 27,062 genes were selected for subsequent analysis (**Supplementary Figure 1A**). The top 2,000 highly variable genes and the top 30 PCs were then chosen (**Supplementary Figures 1B, C**). UMAP clustering identified 14 cell clusters (**Figure 7A**), which were annotated into 12 cell types using marker genes (**Figure 7B**), including macrophages, T cells, AT2s, monocytes, endothelial cells, stromal cells, mast cells, ciliated cells, AT1s, B cells, neutrophils, and proliferating cells. Bubble plots demonstrated the specificity of marker genes for each cell type (**Figure 7C**). Additionally, interactions between cell types were observed in both the COPD and control groups, with weaker interactions in the COPD group compared to the control group (**Figures 7D, E**).

### 3.8 Identification of 7 key cells

**Figure 8:**
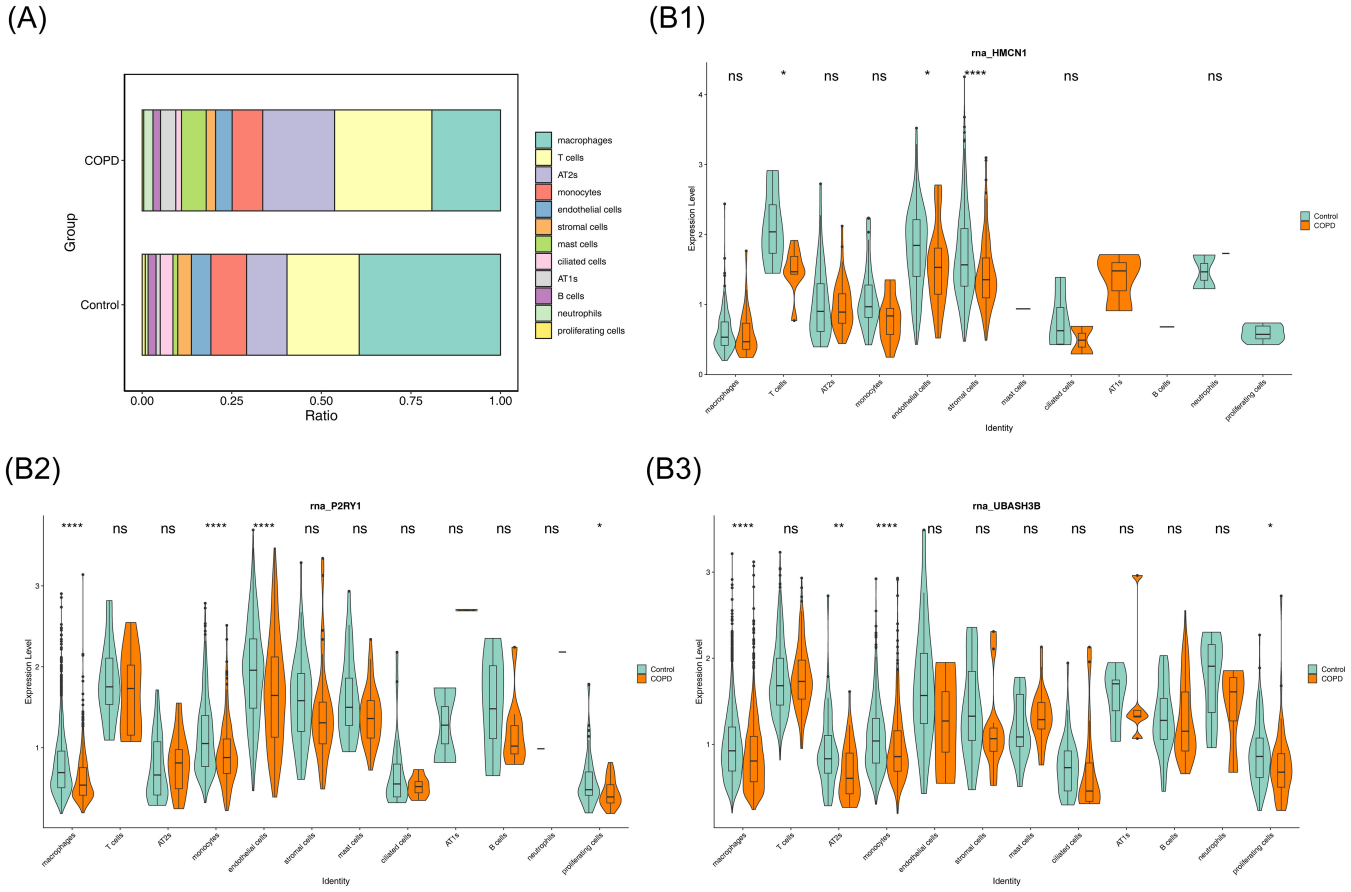
Cellular composition and key gene expression. **(A)** Bar chart showing the proportion of each cell type. **(B)** Violin plot illustrating key gene expression.

The percentage of cell types in COPD and control samples was explored, revealing a decrease in macrophages and an increase in T cells in the COPD group compared to controls (**Figure 8A**). Further analysis of biomarker distribution across the 12 cell types between the groups showed that P2RY1 expression was significantly different in macrophages, monocytes, endothelial cells, and proliferating cells, while UBASH3B exhibited marked differences in macrophages, AT2s, monocytes, and proliferating cells. HMCN1 expression was notably different in T cells, endothelial cells, and stromal cells (p < 0.05). These 7 cell types were selected as key cells for further analysis (**Figure 8B**). Finally, the developmental and differentiation trajectories of the key cells were examined. P2RY1 and HMCN1 expression remained stable during key cell differentiation, whereas UBASH3B expression in macrophages initially decreased, then increased, before stabilizing (**Supplementary Figure 2**). These dynamic changes in biomarker expression over time were clearly illustrated.

### 3.9 Identification and functional analysis of macrophage M1/M2 subsets

**Figure 9:**
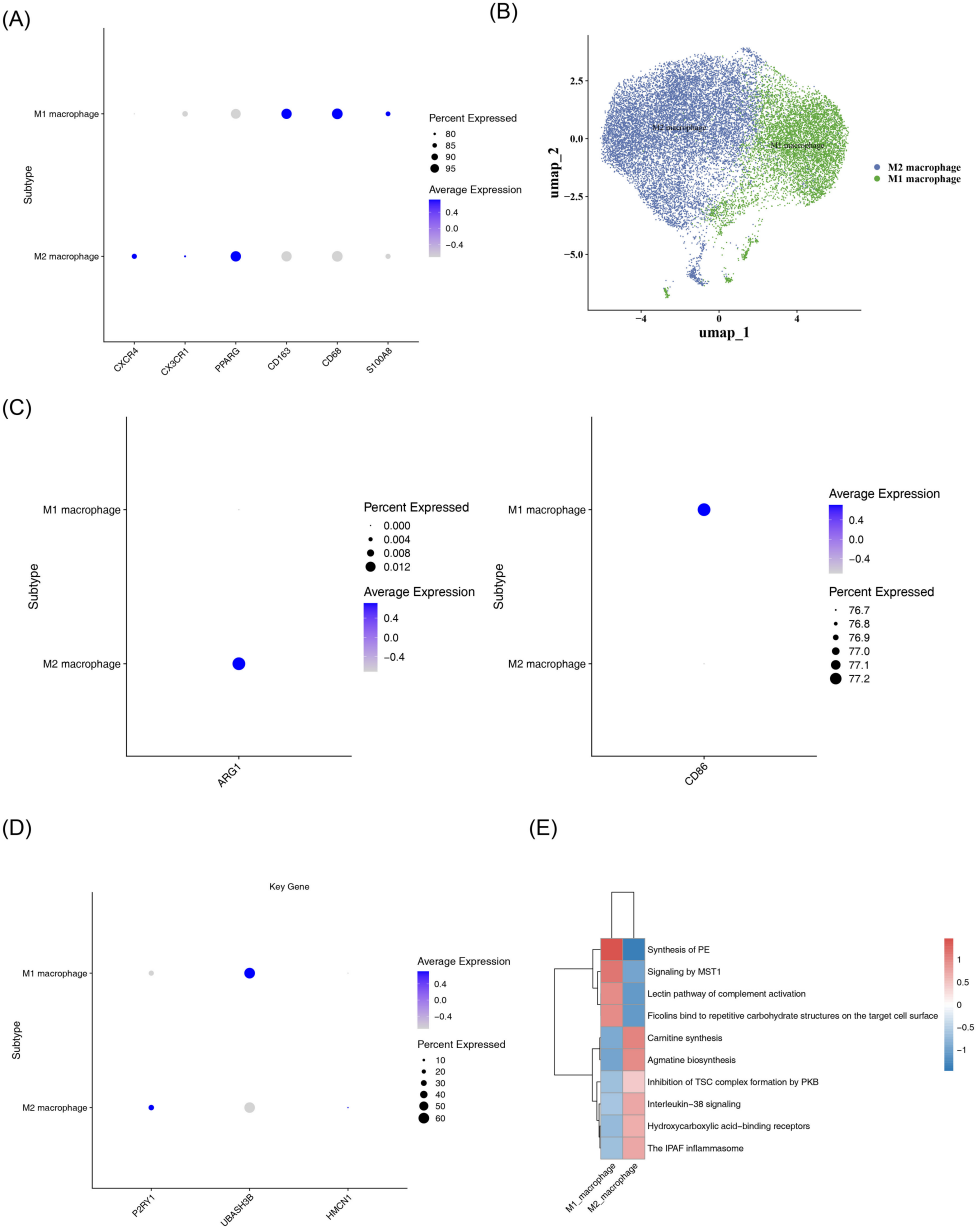
Subtype analysis of macrophages. **(A)** Bubble plot of marker gene expression in M1 and M2 macrophage subtypes. The color gradient, from blue to gray, indicates the average expression from high to low, and the size of the dots corresponds to the percentage of expressed cells. **(B)** UMAP dimensionality reduction visualization plot. It shows the distribution characteristics of M1 (blue) and M2 (green) macrophages, with the axes being umap_1 and umap_2, respectively. **(C)** Bubble plot of polarization marker expression. The left panel shows the average expression of the ARG1 gene in M1 and M2 macrophages; the right panel shows the average expression of the CD86 gene in M1 and M2 macrophages. **(D)** Bubble plot of P2RY1, UBASH3B, and HMCN1 expression in M1 and M2 macrophages. **(E)** Heatmap of enriched pathways in M1 and M2 macrophages. The color ranges from blue (low expression/enrichment) to red (high expression/enrichment), indicating the degree of change.

Macrophages were divided into two subsets, namely M1 macrophages and M2 macrophages, via dimensionality reduction and clustering analysis (**Figures 9A, B**). Expression verification of polarization markers showed that CD86 and ARG1 exhibited differential distribution across different cell types (**Figure 9C**). Expression analysis in the two macrophage subsets demonstrated that (**Figure 9D**): P2RY1 was significantly highly expressed in M2 macrophages, UBASH3B showed relatively high expression in M1 macrophages, while HMCN1 exhibited low expression levels in both cell types. Functional enrichment analysis of the top 10 significantly differential items among different cell types revealed that both M1 and M2 macrophages were significantly enriched in the Phosphatidylethanolamine Synthesis Pathway (**Figure 9E**), suggesting that this pathway might play an important role in both subsets.

### 3.10 RT-qPCR Validation

**Figure 10:**
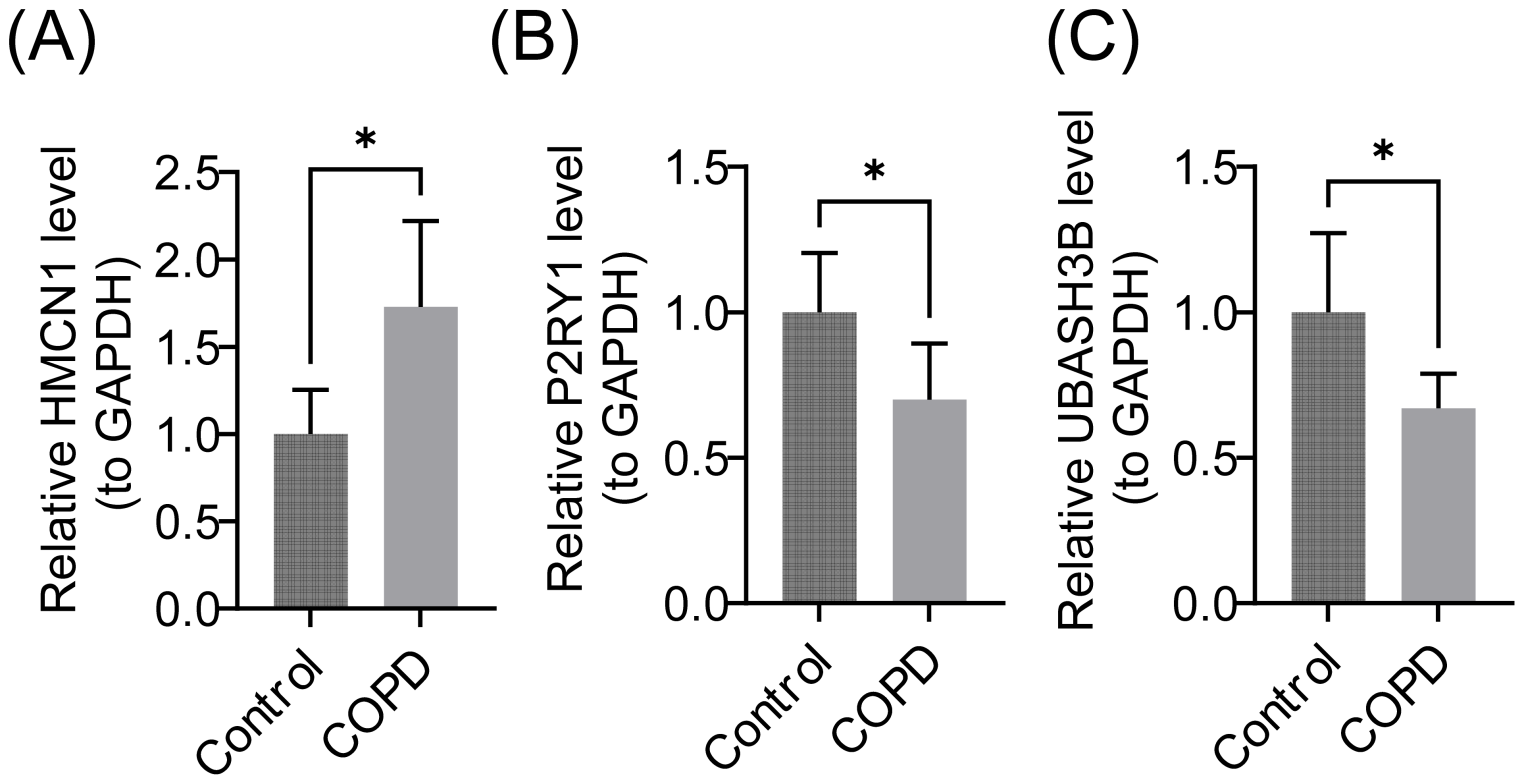
Relative mRNA Expression Levels of Biomarkers. **(A)** HMCN1 gene, **(B)** P2RY1 gene, **(C)** UBASH3B gene. GAPDH (Glyceraldehyde-3-Phosphate Dehydrogenase) was used as the internal reference gene. Data are presented as mean ± standard deviation (SD), * represents P < 0.05.

RT-qPCR results indicated that P2RY1 and UBASH3B were significantly downregulated in the COPD group, while HMCN1 was significantly upregulated (**Figures 10A–C**).

## 4 Discussion

This study identified three genes—P2RY1, UBASH3B, and HMCN1—as biomarkers for COPD through bioinformatics analyses and experimental validation, offering new insights into COPD pathogenesis.

P2RY1, a member of the purinergic receptor P2Y family, is a G-protein-coupled receptor involved in purinergic signaling, regulating processes such as cell proliferation, differentiation, inflammatory responses, and platelet activation (47, 48). In respiratory diseases, P2RY1 activation has been shown to promote airway smooth muscle contraction and recruit inflammatory cells, such as eosinophils, to the airways, exacerbating inflammation in asthma (49). However, no prior studies have linked P2RY1 to COPD. This study is the first to report abnormal expression or dysfunction of P2RY1 in patients with COPD, suggesting it may play a critical role in COPD pathogenesis by regulating airway inflammation (e.g., macrophage activation) or contributing to lung tissue remodeling, thus promoting airway structural changes and functional impairment.

UBASH3B, a ubiquitin-associated domain-containing protein, functions as a scaffold protein regulating tyrosine kinase signaling pathways, particularly those of SRC family kinases. It negatively modulates immune cell activation, including T cells and macrophages. In autoimmune diseases like rheumatoid arthritis, downregulation of UBASH3B increases the secretion of pro-inflammatory cytokines, such as TNF-α, by macrophages, worsening inflammation (50). In oncology, low UBASH3B expression has been associated with tumor cell proliferation and metastasis in lung cancer (51). However, no studies have explored the role of UBASH3B in COPD. This study is the first to reveal abnormal UBASH3B expression in COPD, suggesting it may contribute to COPD pathology by regulating macrophage polarization (e.g., imbalance between pro-inflammatory M1 and reparative M2 phenotypes) or modulating oxidative stress pathways, thereby weakening antioxidant defenses and exacerbating lung parenchymal damage.

HMCN1 (hyaluronan-mediated motility receptor 1) plays a key role in ECM remodeling, cell adhesion, and migration, and regulates the recruitment of inflammatory cells, such as monocytes and macrophages, to injured tissues. In fibrotic diseases, particularly idiopathic pulmonary fibrosis (IPF), elevated HMCN1 expression correlates with fibroblast activation and collagen deposition, promoting pulmonary fibrosis (52). In cardiovascular diseases, HMCN1 is linked to macrophage infiltration in atherosclerotic plaques and plaque instability (53). However, no studies have addressed HMCN1 in COPD. This study is the first to investigate HMCN1’s role in COPD, proposing that it may accelerate disease progression by promoting airway and lung parenchymal fibrosis, exacerbating structural lung damage, or regulating monocyte/macrophage infiltration, thus amplifying chronic inflammation and accelerating lung function decline.

In this study, three key COPD-related genes (P2RY1, UBASH3B, and HMCN1) were identified via bioinformatics analysis, and a nomogram model was constructed. This not only provides a new perspective for deciphering the crosstalk between macrophage polarization and mitochondrial function in COPD, but also reveals that the underlying mechanism of these genes exhibits both differences from and potential associations with previously reported classical COPD markers. Regarding macrophage-related mechanisms, had identified the roles of multiple key molecules: SIGLEC1 is highly expressed in alveolar macrophages of COPD patients and exacerbates lung injury by regulating phagocytic function (54, 55). MARCO, as a key molecule for macrophage homeostasis, shows reduced expression that impairs the ability of macrophages to clear apoptotic cells (56). Targeting the CCL2-CCR2 signaling pathway can alleviate macrophage dysfunction in COPD via the PI3K-AKT axis (57); SOCS1 and HSPB1 may participate in the pathogenesis of COPD by regulating macrophage ferroptosis (58). Additionally, NRF2 activation can restore macrophage function by repairing oxidative metabolic defects (59). Meanwhile, DNA from neutrophil extracellular traps (NETs) promotes NF-κB-dependent autoimmune responses via the cGAS/TLR9 pathway, further exacerbating pulmonary inflammation (60). Notably, both SIGLEC1 and MARCO focus on the intrinsic functions of macrophages. In contrast, the present study found that P2RY1 is strongly negatively correlated with eosinophils (r=-0.71), while UBASH3B is strongly positively correlated with immature dendritic cells (r=0.72). These findings suggest that P2RY1 and UBASH3B participate in COPD pathogenesis through “macrophage-immune cell crosstalk”, which complements classical mechanisms and enriches the understanding of macrophage regulatory networks. In terms of mitochondrial mechanisms, classical mitochondrial dynamics genes such as MFN2 and OPA1 have been proven to be closely associated with cellular senescence and mitochondrial dysfunction in COPD (61). Specifically, downregulation of MFN2 in COPD lung tissue leads to abnormal mitochondrial fission, increased reactive oxygen species (ROS) production, and aggravated lung cell senescence (62). In contrast, HMCN1 identified in this study may indirectly induce mitochondrial dysfunction by affecting the extracellular matrix (ECM)-mitochondrial signaling axis or macrophage metabolic reprogramming (63, 64), thus providing a new direction for research on mitochondrial mechanisms in COPD.Furthermore, the constructed nomogram model offers insights for developing combined therapies targeting the crosstalk between macrophage polarization and mitochondrial function. In the future, experimental studies are required to further verify the expression dynamics of these genes across different COPD phenotypes.

Immunophenotyping revealed significant differences in immune cell abundances between COPD and control samples, with 22 immune cell types showing marked variances between the two groups. This strongly suggests that immune cells play a pivotal role in the pathogenesis of COPD, with immune imbalance potentially being a key factor driving disease progression. For instance, the increase in activated T cells and B cells indicates that the immune response is in a heightened state in COPD, and such hyperactivation could lead to uncontrolled inflammatory responses, further exacerbating lung tissue damage (65). Conversely, alterations in regulatory T cells could disrupt immune homeostasis, hindering the body’s ability to effectively regulate inflammation (66). The positive correlation between UBASH3B and immature dendritic cells may promote immune activation, while P2RY1’s negative correlation with eosinophils could be linked to the modulation of airway inflammation (66). Future studies should delve deeper into these associations to determine how modulating the expression of key genes can regulate immune cell function, offering new targets and strategies for immune-based therapies in COPD.

Regarding gene regulatory networks, the constructed TF-mRNA and miRNA-mRNA networks provide a valuable framework for exploring the upstream regulatory mechanisms of key genes. Several identified TFs and microRNAs (miRNAs) may finely regulate the expression of these genes. For example, certain TFs could bind to the promoter regions of key genes under specific conditions, either promoting or inhibiting their transcription. In contrast, miRNAs may influence the stability and translation of key genes’ mRNA through complementary pairing (67). However, these predictive results require further experimental validation to clarify the authenticity and functionality of these regulatory interactions in COPD, thereby deepening our understanding of the disease’s complex pathogenesis at the gene regulation level.

UBASH3B-Targeted Drugs: Src Kinase Inhibitors (e.g., Bosutinib, Saracatinib):UBASH3B negatively regulates immune cell activation by modulating SRC family kinases, and its downregulation enhances the secretion of pro-inflammatory cytokines (e.g., TNF-α) by macrophages (68). SRC kinase inhibitors have shown efficacy in reducing synovial inflammation in rheumatoid arthritis and mitigating cigarette smoke-induced lung tissue damage and neutrophil infiltration in COPD animal models (69, 70). This study found a positive correlation between UBASH3B and immature dendritic cells, suggesting that it may exacerbate immune imbalance in COPD by promoting antigen presentation. SRC inhibitors could potentially suppress abnormal immune activation by upregulating UBASH3B function. These findings underscore the potential of UBASH3B as an immunotherapeutic target for COPD and support the application of SRC kinase inhibitors, traditionally used in autoimmune diseases, for COPD therapy. HMCN1-Targeted Drugs: ECM Remodeling Modulators (e.g., 4-Methylumbelliferone): HMCN1 facilitates fibroblast activation and collagen deposition by binding to hyaluronic acid, with high expression positively correlating with the degree of pulmonary fibrosis in IPF (71). In COPD, pulmonary parenchymal destruction coexists with abnormal fibrosis, and inhibitors of hyaluronic acid synthesis have been shown to reduce collagen deposition in airway walls and improve lung function (72). This study establishes a direct link between HMCN1 and COPD lung remodeling, supporting the potential use of antifibrotic drugs in treating COPD.

Single-cell RNA sequencing analysis revealed that the proportion of macrophages in COPD samples was approximately 20% lower than in control samples, with abnormal expression of key genes P2RY1 and UBASH3B (p < 0.05). This finding highlights macrophage dysfunction as a critical driver of COPD pathogenesis. Low P2RY1 expression relieves the inhibition of pro-inflammatory cytokine release (e.g., IL-6, TNF-α) in macrophages by downregulating the cAMP signaling pathway, leading to M1 polarization imbalance and exacerbating airway inflammation and lung tissue damage (73, 74). UBASH3B shows a dynamic expression pattern during macrophage differentiation, with early downregulation triggering abnormal activation of immature macrophages via the SRC kinase-ITAM signaling pathway, promoting excessive CD4+ T cell proliferation. Although expression rebounds during disease progression, it is insufficient to restore immune homeostasis, ultimately resulting in a “pro-inflammatory-immune” positive feedback loop (75). Concurrently, the reduction in macrophage count in COPD impairs M2 polarization-mediated secretion of TGF-β and VEGF, hindering lung repair. High expression of HMCN1 in stromal cells attempts to compensate by recruiting monocytes via hyaluronic acid, but the abnormal activation of fibroblasts exacerbates collagen deposition, leading to a “repair-fibrosis” imbalance (76, 77). In summary, macrophages in COPD exhibit triple abnormalities of “reduced number—polarization imbalance—repair dysfunction.” Future research should focus on the dynamic heterogeneity of macrophages and integrate gene regulatory networks and drug prediction approaches to develop precision therapies based on macrophage reprogramming.

This study has several limitations. The data in this study were mainly derived from public databases, with a relatively limited sample size. A small sample size may reduce statistical power and increase the risk of false-negative or false-positive results. Secondly, the samples were obtained from different research projects, and differences exist among various datasets in terms of sample collection, processing, and experimental conditions. Such heterogeneity may introduce batch effects, thereby affecting the accuracy of gene expression analysis. Additionally, the lack of clinical staging information and detailed phenotypic characteristics of COPD patients may easily lead to increased sample heterogeneity, which could impact the stability and reliability of the research results. Variations in sample collection, processing, and experimental conditions across different studies could affect the research outcomes. Moreover, while bioinformatics offers valuable insights, conclusions drawn solely from data lack sufficient experimental validation.In addition, while bioinformatics, as an efficient data mining tool, can reveal gene associations and potential mechanisms, the conclusions of this study derived from data have not yet been supported by direct evidence from *in vitro* cellular experiments or *in vivo* animal experiments, which limits the persuasiveness of the mechanism explanation. In the future, we plan to enroll samples of COPD patients through multi-center and staged approaches, and conduct stratified analysis based on pulmonary function parameters. This will help expand the clinical cohort and improve the generalizability of the study. Meanwhile, we will integrate cell biology, molecular biology, and animal experiments to validate and expand the research findings. Specific efforts will include: using THP-1 or alveolar macrophage models to explore the effects of genes on cell polarization and mitochondrial function; establishing a cigarette smoke-exposed mouse model to evaluate the effect of gene knockout; analyzing the regulatory relationships between candidate genes and COPD-related pathways; and confirming the interaction between HMCN1 and extracellular matrix (ECM) proteins such as type I collagen via CoIP/MS.These endeavors aim to deepen the research on COPD-related mechanisms and provide a solid basis for the precise diagnosis and treatment of COPD.

## 5 Conclusion

This study identified three genes (P2RY1, UBASH3B, HMCN1) associated with COPD, related to mitochondria and macrophage polarization, through differential expression analysis, enrichment analysis, machine learning, immune infiltration analysis, and other bioinformatics methods. These findings were validated in patients with later-stage COPD, offering new key genes for the diagnosis and treatment of COPD and providing deeper insights into the molecular mechanisms of the disease.

## Data Availability

The datasets presented in this study can be found in online repositories. The names of the repository/repositories and accession number(s) can be found in the article/**Supplementary Material**.
