# Supplementary material for: Bioinformatics identification of mitochondria and macrophage polarization-related genes in COPD and their potential mechanisms
Source: Front Immunol. 2025 Oct 8;16:1675292. doi: 10.3389/fimmu.2025.1675292 (PMC12540082; doi:10.3389/fimmu.2025.1675292)
Supplement: Supplementary file 1 [file Supplementaryfile1.docx]

Supplementary Material

# Supplementary Figures

**
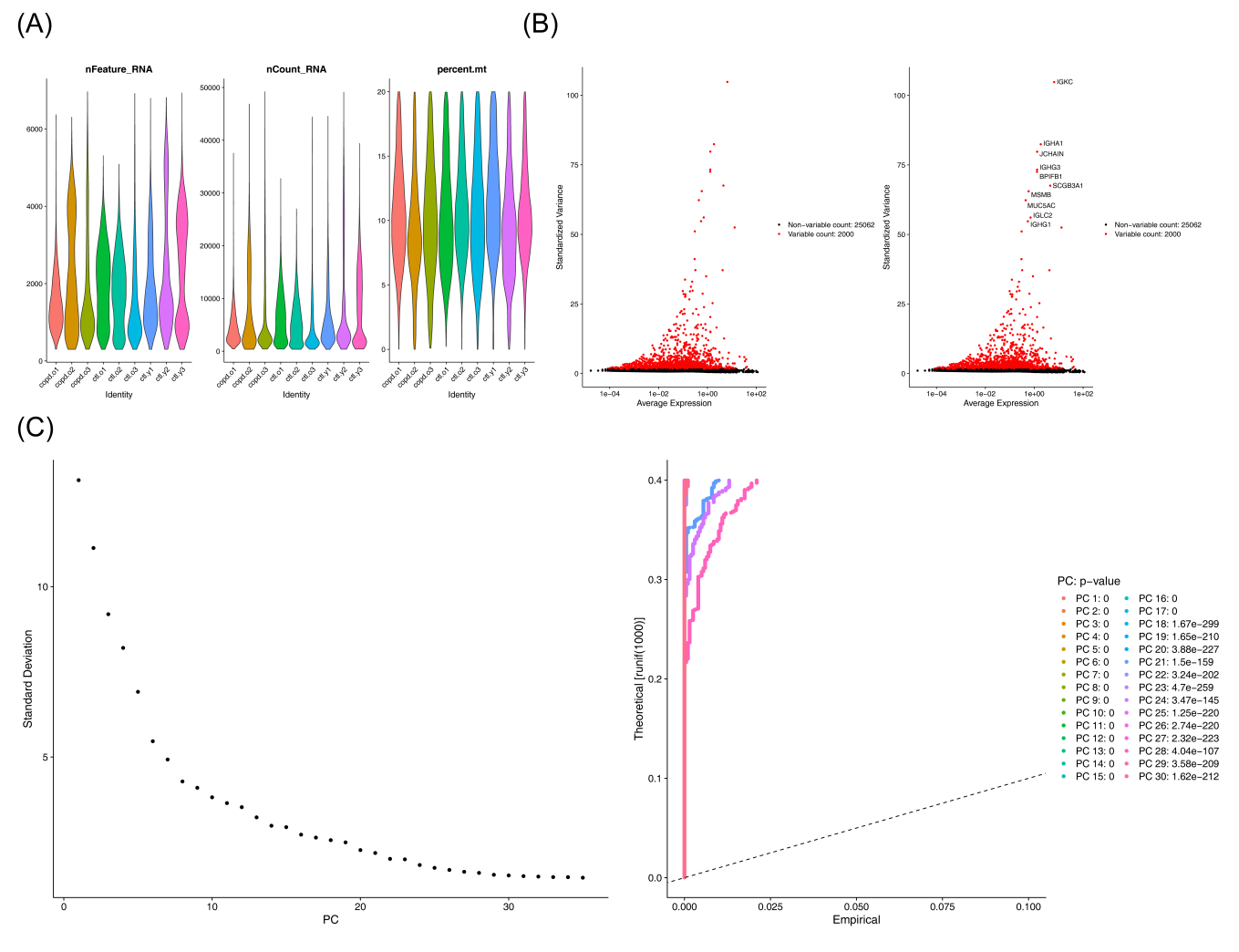
**

**Supplementary Figure 1.** Quality control, highly variable gene screening, and principal component analysis of single-cell RNA sequencing data. **(A)** Violin plots showing the distribution of quality control metrics across different cell populations. From left to right, they are nFeature_RNA (the number of genes detected in a single cell), nCount_RNA (the total UMI count of a single cell), and percent.mt (the proportion of mitochondrially derived genes, reflecting the degree of mitochondrial contamination). **(B)** Scatter plots of highly variable gene identification results. With "average gene expression" as the x-axis and "normalized variance" as the y-axis, red dots represent the screened variable genes (the left panel shows the distribution of all genes, and the right panel labels the top 10 variable genes such as HBA1, HBB, etc.), while black dots represent non-variable genes. **(C)** Evaluation results of principal component analysis (PCA). The left panel is a "principal component (PC) - standard deviation" scatter plot (elbow plot), which is used to preliminarily determine the number of key principal components to be retained; the right panel is a QQ plot from PCA permutation test, showing the distribution correspondence between "theoretical p-values" and "empirical p-values", and the significant p-values of each PC are listed on the right to screen for PCs with statistical differences.

**
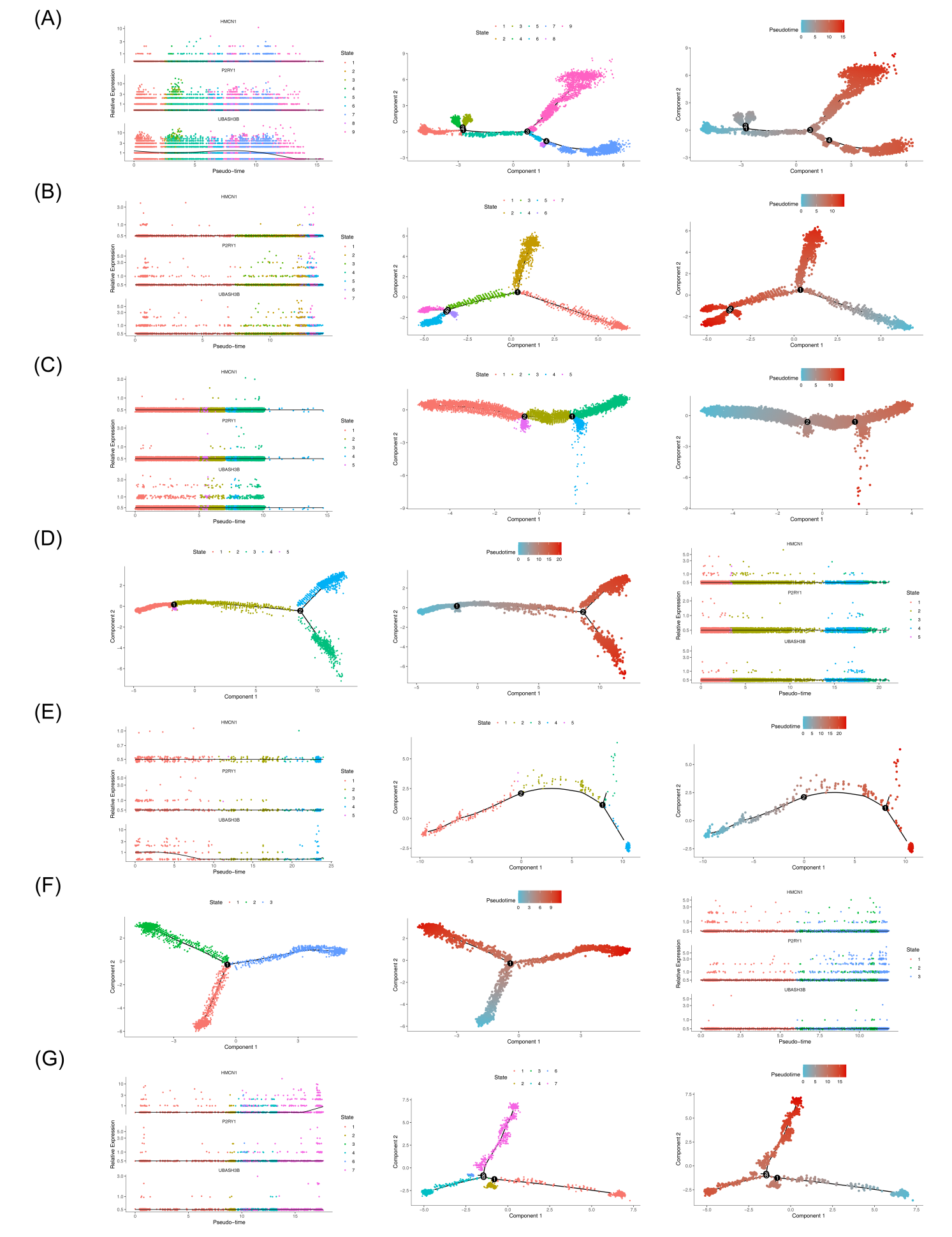
**

**Supplementary Figure 2.** Pseudotime analysis of different key cell types. **(A-G)** represent the single-cell pseudotime analysis results of macrophages, monocytes, T cells, AT2s, proliferating cells, endothelial cells, and stromal cells in sequence. Each group contains three panels: the left panel is a pseudotemporal gene expression heatmap, showing the expression changes of biomarkers during the pseudotime process; the middle panel is a dimension-reduced scatter plot of the cellular pseudotime trajectory; the right panel is a pseudotime-colored pseudotime trajectory plot, where the color from blue to red represents pseudotime from early to late, reflecting the distribution of cells along the differentiation trajectory.

# Supplementary Tables

**Supplementary Table 1** Summary of the included datasets and their baseline characteristics.

| Data | sample number | source name | submit date | age | | | | Country | GPL | Gender |
| --- | --- | --- | --- | --- | --- | --- | --- | --- | --- | --- |
|  |  |  |  | Min. | Median | Mean | Max. |  |  |  |
| GSE151052 | COPD:Control (77:40) | Lung tissue | May 22 2020 | 39.00 | 55.00 | 56.97 | 77.00 | Canada | GPL17556 |  |
|  |  |  |  |  |  |  |  |  |  |  |
| GSE106986 | COPD:Control (14:5) | Lung tissue | Feb 08 2018 | 44.00 | 69.00 | 66.84 | 79.00 | Germany | GPL13497 | Female:Male (7:12) |
| GSE171541 | COPD:Control (3:6) | Lung tissue | Dec 28 2022 | 27 | 63 | 55 | 75 | China | GPL24676 |  |

**Supplementary Table 2** Primer sequences used in qRT-PCR.

| Primer name | Sequence |
| --- | --- |
| P2RY1 F | CCGTCTCCTCGTCGTTCAAA |
| P2RY1 R | ACGTACAAGAAGTCGGCCAG |
| UBASH3B F | TGGACGTGCTCCTCTCCAT |
| UBASH3B R | TGGGAGAATAACCAACCAGTCAC |
| HMCN1 F | GTGGGGAGTGAGAGAAAAGCC |
| HMCN1 R | TAGTGGGTGGGGCATCATCTA |
| GAPDH F | CGAAGGTGGAGTCAACGGATTT |
| GAPDH R | ATGGGTGGAATCATATTGGAAC |
